# Effects of habitat edges on vegetation structure and the vulnerable golden-brown mouse lemur (*Microcebus ravelobensis*) in northwestern Madagascar

**DOI:** 10.1186/s12898-020-00337-z

**Published:** 2020-12-17

**Authors:** Bertrand Andriatsitohaina, Daniel Romero-Mujalli, Malcolm S. Ramsay, Frederik Kiene, Solofonirina Rasoloharijaona, Romule Rakotondravony, Shawn M. Lehman, Ute Radespiel

**Affiliations:** 1 Ecole Doctorale Ecosystèmes Naturels (EDEN), University of Mahajanga, Mahajanga, Madagascar; 2 Institute of Zoology, University of Veterinary Medicine Hannover, Buenteweg 17, 30559 Hannover, Germany; 3 Department of Anthropology, University of Toronto, Toronto, Canada; 4 Mention Sciences de la Vie et de l’Environnement, Faculté des Sciences, de Technologies et de l’Environnement, University of Mahajanga, Mahajanga, Madagascar

**Keywords:** Habitat loss, Edge effects, Habitat choice, Vegetation structure, Abundance, Mouse lemur, Depth of edge influence, DEI, *Microcebus ravelobensis*, Madagascar

## Abstract

**Background:**

Edge effects can influence species composition and community structure as a result of changes in microenvironment and edaphic variables. We investigated effects of habitat edges on vegetation structure, abundance and body mass of one vulnerable *Microcebus* species in northwestern Madagascar. We trapped mouse lemurs along four 1000-m transects (total of 2424 trap nights) that ran perpendicular to the forest edge. We installed 16 pairs of 20 m^2^ vegetation plots along each transect and measured nine vegetation parameters. To determine the responses of the vegetation and animals to an increasing distance to the edge, we tested the fit of four alternative mathematical functions (linear, power, logistic and unimodal) to the data and derived the depth of edge influence (DEI) for all parameters.

**Results:**

Logistic and unimodal functions best explained edge responses of vegetation parameters, and the logistic function performed best for abundance and body mass of *M*. *ravelobensis*. The DEI varied between 50 m (no. of seedlings, no. of liana, dbh of large trees [dbh ≥ 10 cm]) and 460 m (tree height of large trees) for the vegetation parameters, whereas it was 340 m for *M. ravelobensis* abundance and 390 m for body mass, corresponding best to the DEI of small tree [dbh < 10 cm] density (360 m). Small trees were significantly taller and the density of seedlings was higher in the interior than in the edge habitat. However, there was no significant difference in *M. ravelobensis* abundance and body mass between interior and edge habitats, suggesting that *M. ravelobensis* did not show a strong edge response in the study region. Finally, regression analyses revealed three negative (species abundance and three vegetation parameters) and two positive relationships (body mass and two vegetation parameters), suggesting an impact of vegetation structure on *M. ravelobensis* which may be partly independent of edge effects.

**Conclusions:**

A comparison of our results with previous findings reveals that edge effects are variable in space in a small nocturnal primate from Madagascar. Such an ecological plasticity could be extremely relevant for mitigating species responses to habitat loss and anthropogenic disturbances.

## Introduction

Forest loss leading to fragmentation is the main consequence of human activities in forest ecosystems. The most obvious impact of this process is an increase in the number of patches coupled with a decreasing patch size, increasing isolation and especially an increase of edge effects and of the proportion of edge habitat in a given patch [1, 2]. Three types of ecological edge effects were previously described [3]: (1) direct abiotic effects (i.e. changes in microclimate such as in solar radiation, temperature, humidity, or wind speed), (2) indirect biological effects which result from abiotic changes (e.g., changes in plant composition due to increased solar radiation or a change in the abundance of animal species due to changes in food availability), and (3) indirect biological effects due to changes in species interactions, e.g. in competition, [3]. Proximity to forest edges can alter fauna and flora in different ways. In principle, there are three different types of ecological edge responses: (1) “positive” edge responses (i.e. abundance increases near edges), (2) “neutral” edge responses (i.e. abundance does not change with increasing distance from edge), and (3) “negative” edge responses (i.e. abundance decreases near edges) [4].

A variety of vegetation changes have been reported as responses to proximity to forest edges. For example, interior trees had a greater dbh (defined as diameter at breast height) than edge trees in a fragmented rainforest in Costa Rica [5]. Similar results were reported in Brazilian fragmented landscapes, with the basal area of trees being lower in proximity to the edge [6]. In contrast, the density of some woody species was higher near the edge in Morogoro Region, Tanzania [7]. A study from the Thiaki Creek Nature Reserve, Australia, reported that the probability of survival of seedlings and trees was higher closer to intact forest than at the edge [8, 9], and that seedling survival was correlated to edaphic and microenvironment variables [10]. Thus, vegetation responses to edge effects can be complex and vary depending on local conditions, plant species traits, and the depth and intensity of edge effects [4].

Positive, neutral, and negative edge responses have also been reported for various vertebrate taxa. For example, some mammals species, such as primates and some rodents, had higher abundance metrics in habitat edges compared to interior forest habitats [6, 11]. In contrast, some marsupial, bird or other rodent species showed higher population densities far away from edges [6, 12, 13]. No spatial variations in edge-related abundance were observed in some Neotropical primate species [5, 11]. However, edge responses of mammals are not always stable across landscapes [6], an insight which resulted from a long-term fragmentation study in the Amazon region and was incorporated in the “landscape-divergence-hypothesis” [14]. This hypothesis states that as a consequence of high local landscape and weather dynamics, sites from different landscapes will diverge more over time (in species composition and possibly ecosystem functioning) than sites from the same landscape. However, it is not known if this variability in edge responses in time and space occurs in other continents and phyla.

Many studies in different biomes and continents have shown that the depth of edge influence (DEI) can differ largely between parameters [15]. The DEI is defined as the distance at which biotic or biotic parameters change considerably from the edge to the interior of the forest. However, determining the DEI is not a simple task, as habitat edges may affect plants and animals in various non-linear ways [16]. Previous authors used different qualitative and quantitative approaches to estimate the DEI, e.g., [15, 17–20]. Ewers and Didham formalized the first mathematical method to determine the DEI, which is based on a relative comparison of the accuracy of different mathematical functions to describe continuous response functions for any biotic or abiotic variable across ecological boundaries [21] and shall be employed in this study on a small Malagasy lemur species.

Madagascar is one of the world’s most important biodiversity hotspots and is experiencing widespread changes in forest cover due to climate change, forest loss, and habitat fragmentation [22]. Forest loss has resulted in a reduction of the island’s forest cover by 44% between 1953 and 2014 [23]. Remaining forests exist primarily as landscapes of forest fragments of varying size and shape surrounded by a matrix such as grasslands or agricultural fields. These forest landscapes represent an ideal natural laboratory for studying edge effects on plants and animals, providing an important opportunity to model extreme levels of deforestation processes. In turn, results from research on Madagascar can inform similar work in other tropical regions not yet subject to the same extremes of forest loss and fragmentation. Clearly, understanding how plants and animals respond to edge effects is crucial for conservation planning [24].

It was already shown in some Malagasy rainforest sites that edge effects can affect vegetation structure and the abundance and distribution of animal species such as birds or lemurs e.g., [25–28]. Extant lemurs are a highly threatened mammalian clade that comprises more than 100 described taxa which are endemic to Madagascar [29, 30]. Studies from rainforest habitats described three types of edge responses in lemurs: edge tolerance, neutral, or negative edge responses, e.g., [26–28]. Moreover, these studies noted that tree height and dbh were smaller at the edge compared to the interior of forest [28].

However, there are only few data on how edge effects influence the vegetation in the tropical dry forests in western Madagascar [31, 32] and also on how animals respond to edge effects in these forests [17]. In the dry deciduous forests of northwestern Madagascar, the golden-brown mouse lemur (*M. ravelobensis*) was found to be more abundant in edge habitats than in the forest interior in one part of the Ankarafantsika National Park; this was not the case for the grey mouse lemur (*M. murinus*) which lives in sympatry with golden-brown mouse lemurs at this site [33]. Moreover, female *M. ravelobensis* weighed more in edge habitats than in the interior, which was not observed in *M. murinus*. At the same study site, a higher density of stems and seedlings was found in the forest interior than close to forest edges, but there was no difference of tree dbh between the two habitats [31, 32]. Larger scale landscape comparisons of edge effects within the same biome, however, are still missing from Madagascar. They would allow more generalized insights into edge effects, which could result in more nuanced conservation management suggestions.

Our study focused on the vulnerable *M. ravelobensis* which has a limited geographic distribution in the dry deciduous forests of northwestern Madagascar [34, 35]. *M. ravelobensis* is a nocturnal, small-bodied (~ 60 g), arboreal, omnivorous solitary forager and occurs in partial sympatry with *M. murinus* in some but not all forests of northwestern Madagascar [36, 37]. *M. ravelobensis* forms mixed‐sex sleeping groups and sleeps in tree-holes but also in dense tangles of lianas, leaves or self-built nests [38, 39]. Previous studies reported certain microhabitat preferences of this species, such as habitats containing higher density of trees with many lianas and a higher cover of the herb layer, and habitats closer to surface water or at lower altitudes [36, 37, 40, 41]. Most recently, significant regional differences were revealed in the abundance of *M. ravelobensis* across its distribution [37]. Whether these differences correspond to differences in edge responses, however, is not yet known.

The aims of this study were to use ecological modelling techniques to evaluate edge effects (1) in vegetation structure, (2) on the distribution and body mass of *M. ravelobensis*, and (3) on the spatial variability of these parameters based on comparisons with previous studies from a different study region in northwestern Madagascar.

In particular, we addressed the following questions:

1. Does the vegetation structure change systematically from the forest edge to the interior of the forest? If so, in which way?
2. Does *M. ravelobensis* abundance and body mass change from the forest edge to the interior? If so, in which way?
3. Is there a relationship between edge-related vegetation changes and changes in *M. ravelobensis* abundance or body mass?
4. Are edge responses in *M. ravelobensis* stable between sites (locally and regionally)?

## Methods

### Study site and period

**Fig. 1:**
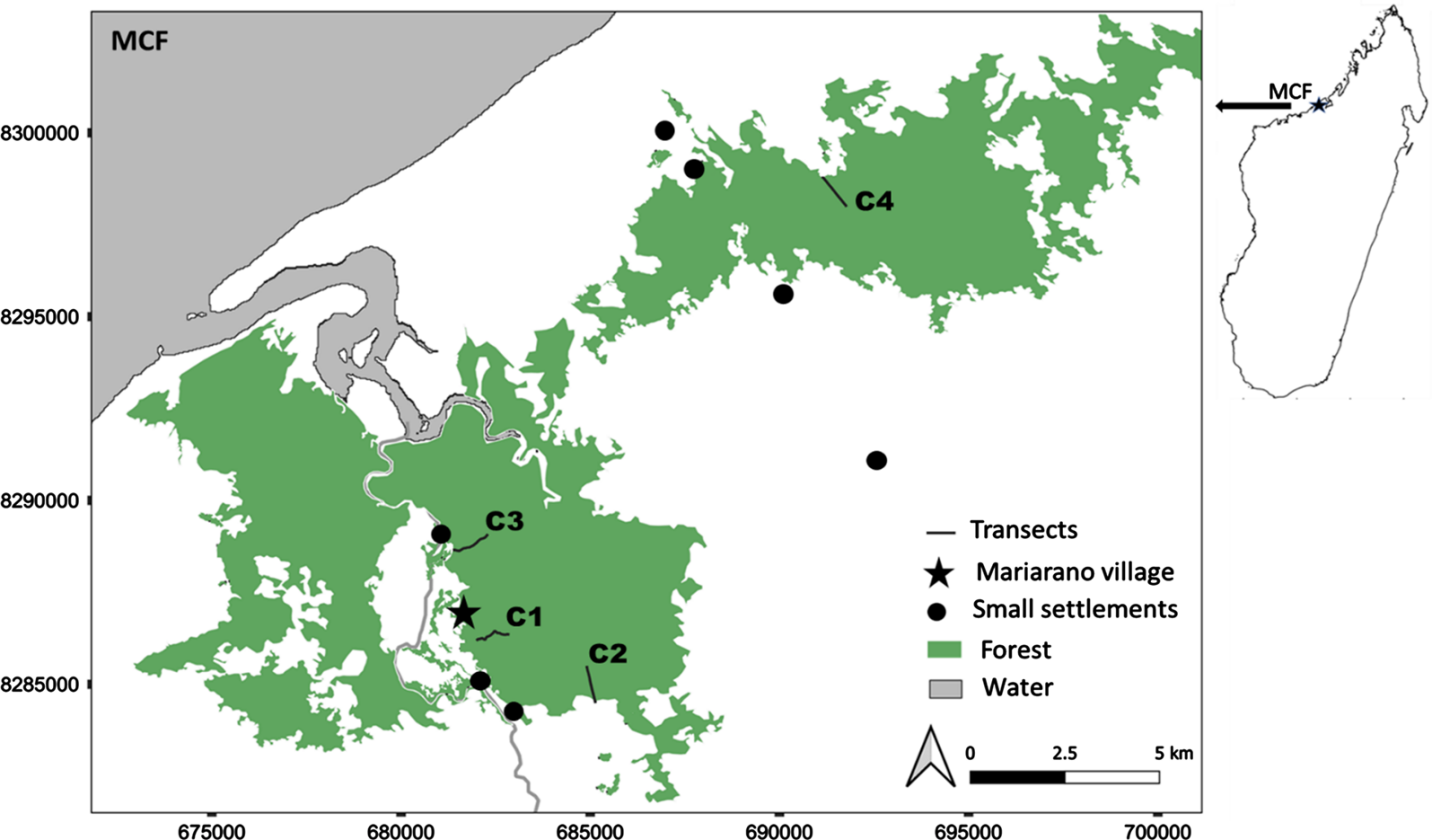
Map of Mariarano Classified Forest (MCF) showing the study area and the location of all four study transects (C1, C2, C3 and C4) in relation to settlements and forest edge. The inset at the top right shows the location of the study site (star) within Madagascar (map drawn by: B. Andriatsitohaina)

This study was conducted in the Mariarano classified forest (MCF), located near the coast (15° 24′ S, 46° 44′ E, 80 km northeast of Mahajanga) and next to the village Mariarano at an altitude of 20-90 m a.s.l. (Fig. 1) in NW Madagascar. The MCF encompasses 147,200 ha of partially fragmented dry deciduous forest managed by local community committees known as Vondron’Olona Ifotony (VOI). The areas adjacent to the forest belong to the most productive agricultural land in Mahajanga II district [42]. We performed field work in June–July 2017 (transect C1) and in mid May–mid August 2018 (transects C2–C4). All fieldwork protocols were reviewed and approved by the Institute of Zoology, University of Veterinary Medicine Hannover, Germany and the University of Toronto, Canada. This study was approved by the Ministère de l’Environnement, de l’Ecologie et des Forêts (permit numbers 151/17/MEEF/SG/DGF/DSAP/SCB.Re and 82/18/MEEF/SG/DGF/DSAP/SCB.Re).

### Data collection

We established four transects of 1000 m length each of which ran perpendicular to the forest edge. These transects bordered different matrix types: road with bushes across (C1), savannah with low density of palm trees (*Bismarckia nobilis* or *Satrana* as local name) (C2), rice fields with mango trees (C3), and savannah with a high density of palm trees (*B. nobilis*) (C4). Mouse lemurs were captured systematically three times over a 2-week period along each transect by means of Sherman traps that were baited with banana and installed in pairs every 10 m (one trap on either side of the transect) at 0.5 – 2.0 m height above ground. A total of 2424 trap nights (202 traps per trapping session per transect) were conducted across all four transects. After the identification of species by means of phenotypic and morphometric characterization (following [34]), all captured individuals of *M. ravelobensis* were sexed, aged (juvenile/adults), individually marked (by ear biopsies), and weighed. All handling procedures were quick and therefore performed without the use of anesthesia as in many other studies before [e.g., 33–35]. We classified an individual as adult if the body mass was higher or equal to 41 g and in view of some other morphometric parameters (e.g. lower leg length, hindfoot length). Finally, all individuals were released at dusk at the same place where they had been trapped on the day of capture. We determined abundances of *M. ravelobensis* across each transect by calculating the number of individuals caught (without recapture) per 100 m transect length (= 20 trap locations). The data from 2017 and 2018 were combined in all analyses given the considerable seasonal overlap between both years, the lack of systematic change in trappability of mouse lemurs across the dry season in northwestern Madagascar [36], and consistent trapping results across several years along two of the transects (C1, C3, Radespiel unpubl. data). A total of 226 individual *M. ravelobensis* (100 males, 126 females) were trapped across the study period. In contrast, only one individual of *M. murinus* was trapped on one transect (C2) during the entire study period (Fig. 1).

We installed 16 pairs of vegetation plots along each transect (2 m × 10 m, W x L), oriented perpendicularly to either side of a transect at pre-set distances from the forest edge to the interior (at 0 [edge], 20, 40, 60, 80, 100, 200, 250, 300, 400, 500, 600, 700, 800, 900, and 1000 m) according to [31]. In each plot, vegetation measurements focused on dendrometrics and estimated densities of woody stems by counting the number of seedlings (1–100 cm in height), saplings (101–250 cm in height), trees (height > 250 cm) and lianas (with dbh ≥ 2.5 cm). We measured the height and dbh of all trees rooted in a plot. From these data, we calculated the total number of trees (categorized into small: dbh < 10 cm, and large: dbh ≥ 10 cm), seedlings, saplings and lianas per double plot (40 m^2^) at each sampling distance from the edge for each transect and then calculated the overall mean density across the four transects. Similarly, we calculated the mean height and dbh of small and large trees (per 40 m^2^) at each sampling distance across all four transects. These mean values were used to determine the DEI according to methods in Ewers and Didham [21].

We calculated the mean vegetation parameters per 20 m^2^ plot at each distance from edge for each transect (= four mean values for each sampling point) for the comparison of habitat parameters between the edge habitat and the interior of the forest. The values for all vegetation parameters were also summarized as means/100 m section along each transect to prepare the vegetation dataset for comparisons with the animal dataset.

### Data analyses

Following Ewers and Didham [21] we calculated and compared the fit of four different mathematical models, linear (Eq. 1), power (Eq. 2), logistic (Eq. 3), and unimodal (Eq. 4) to the data to evaluate how the relationship between the distance to the edge and the test parameters could be best formalized. Support for the linear model would indicate a lack of a finite impact of the edge whereas the three other models can be used to determine the depth of edge influence (DEI).

The chosen test parameters were:

- vegetation parameters: density, dbh and height of small trees (dbh < 10 cm); density, dbh and height of large trees (dbh ≥ 10 cm); density of saplings; density of seedlings and density of lianas.
- Animal parameters: *M. ravelobensis* abundance and body mass.

Based on Ewers and Didham, the formulas for the four used mathematical models were:

- Linear
  1 $${\text{n}}_{x} = \, \beta_{ 1} + \, \beta_{ 2} {\text{x }} + \, \varepsilon$$
- Power
  2 $${\text{n}}_{x} = \, \beta_{ 1} *e^{{\beta 2*{\text{x}}}} + \, \varepsilon$$
- Logistic
  3 $$n_{x} = \, Y_{\hbox{min} } + \, \left( {Y_{\hbox{max} } - \, Y_{\hbox{min} } } \right)/\left( {1 \, + e^{{(\beta 1 \, {-}x)\beta 2}} } \right) \, + \varepsilon$$
- Unimodal
  4 $${\text{n}}_{x} = {\text{ Y}}_{ \hbox{min} } + \, \left( {\left( {{\text{Y}}_{ \hbox{max} } - {\text{ Y}}_{ \hbox{min} } } \right)/\left( { 1 { } + {\text{ e}}^{{(\beta 1 { }{-}{\text{ x }} + \beta {\text{x2}})\beta 2}}_{ 3} } \right)} \right) \, + \varepsilon$$

where n is the mean of given response variable (see above), Y_max_ and Y_min_ are the maximum and minimum values of the response variable, β_1_, β_2_ and β_3_ are constants, x is the distance to edge, and ε is an error term.

The process was repeated 1000 times in R (version 3.6.0). The best fit of each of the four mathematical models was compared for each parameter using an information theoretic approach and calculated Akaike’s Information Criterion with a correction for small sample size (AICc) and R^2^, with lowest value of AICc and the highest R^2^ indicating the best model [43, 44].

We determined the DEI (defined as the edge distance over which changes in the response variable can be detected) by calculating the first and second derivative of the best model (for functions see above). The first derivative describes the local minimum or maximum of the rate of change in the response variable. The second derivative enabled calculation of the DEI as the distance between the two first inflection points in the curve describing the parameter under study [21, 45]. Based on the second derivative curve, we used the “inflection” package (vs. 1.3.5) and employed the Extremum Surface Estimator ESE method by means of the findipiterplot() command in R (version 3.6.0) to determine the distance between the two first inflection points in the curve, i.e., the DEI [46].

We subsequently compared the data for each vegetation parameter (one value per double plot per transect) between respective “edge habitat” and the “interior habitat” statistically by means of generalized linear models (GLMs) using the R package “lme4” (version 1.1-21). Similarly, the abundance (one value per 100 m per transect) and adult body mass of *M. ravelobensis* captured in “edge habitat” and “interior habitat” of the forest were compared by means of GLMs. The “edge habitat” and “interior habitat” were determined for each vegetation and animal parameter separately based on the respective DEI which was “buffered” to both sides. “Buffering” was achieved by first considering the respective DEI for each parameter and then excluding the data-point closest to that DEI in both directions (i.e., towards edge and towards interior) to account for the disjunct spatial data collection scheme in vegetation plots and the representation of all animal data in fixed 100 m segments.

Next, the DEI estimates derived for the different vegetation parameters were compared to the DEI inferred for *M. ravelobensis* abundance and adult body mass to explore which vegetation parameter corresponds best to edge-related variations in animal abundance and body mass. To evaluate this potential relationship between animals and vegetation structure further, we calculated one value per 100 m per transect for each animal and vegetation parameter, and performed regression analyses using lm() function with *M. ravelobensis* abundance and adult body mass as dependent variables and the vegetation parameters on the other side as independent variables.

For all analyses, normality of all variables was tested using the Shapiro–Wilk’s test [47]. If a variable was not normally distributed, it was log‐transformed (number of large trees, number of saplings, number of seedlings and number of lianas for regression analysis) and Q–Q plots visualization of the data were used before and after transformation to ensure improvement. Significance level was set at α = 0.05 and a statistical trend was indicated by 0.1 > p > 0.05.

## Results

### Effects of habitat edges on vegetation structure

**Fig. 2:**
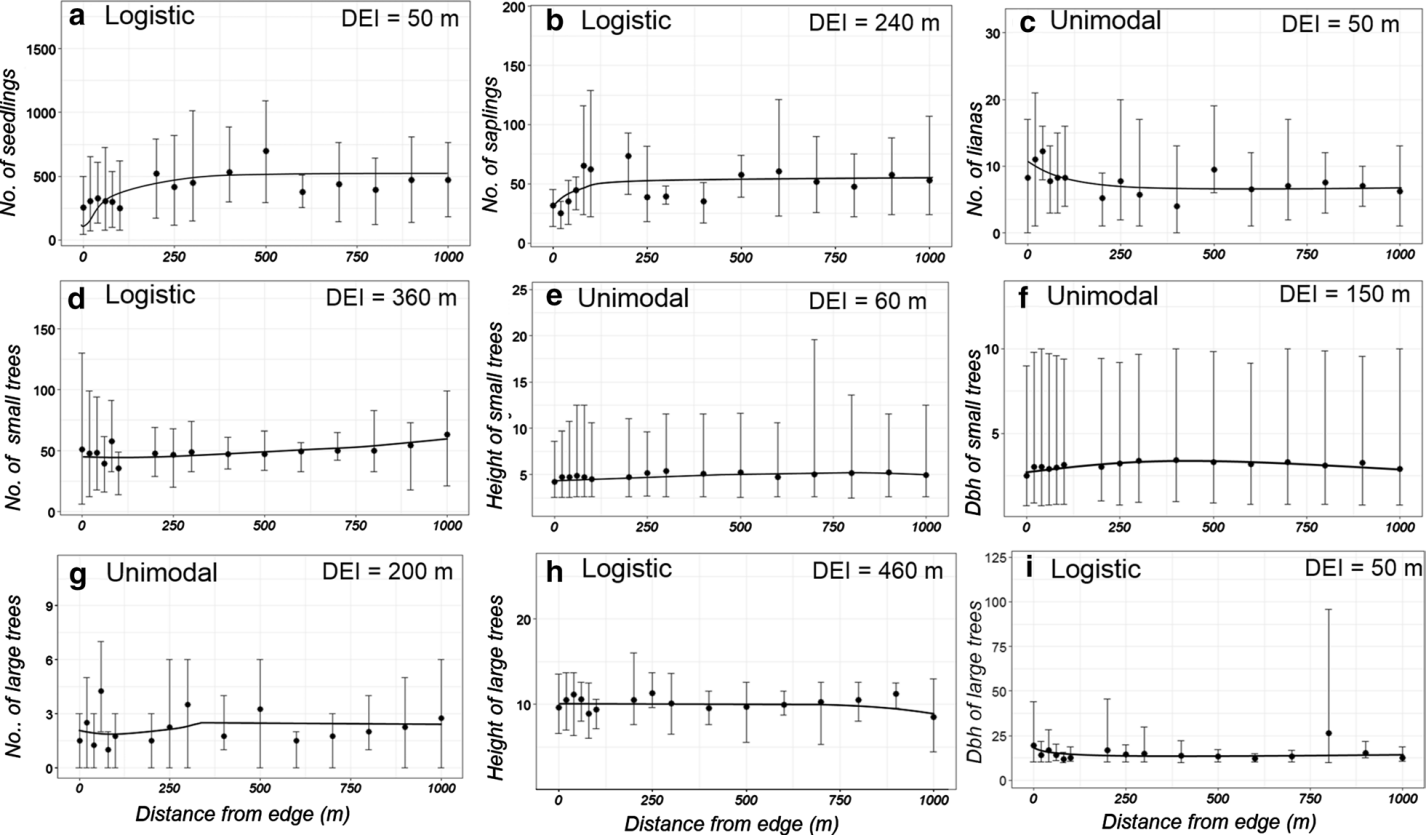
Illustration of the best function (logistic and unimodal) and averaged values of all nine vegetation parameters across the length of the transects (**a**–**i**) and their respective depth of edge influence DEI. Mean values (dots) are shown together with minimum and maximum values (whiskers). Lines represent the best fit of the distribution of all nine vegetation parameters across distance from edges

In total, we estimated the density and size of 26,019 seedlings; 3114 saplings; 489 lianas; 3143 small trees; and 139 large trees in the 128 installed plots (Additional file 1: Table S1). Across the nine vegetation variables, the logistic function best explained the edge responses in five vegetation variables (seedlings, saplings, dbh of large trees, number of small trees, and height of large trees), whereas the unimodal function best explained the variation in the remaining four variables (number of lianas, number of large trees, height of small trees and dbh of small trees, Additional file 1: Table S2 and Fig. 2). The power function and the linear function did not explain edge responses in any variable. Although R^2^-values of the best models varied considerably (range 0.017–0.549), they explained more than 10% of the variation in the data for seven of nine vegetation parameters (Fig. 2a–f, h), but not for the number and dbh of large trees (R^2^ < 0.1, Fig. 2g, i, Additional file 1: Table S2). The DEI, derived from the second derivative of the best models, varied across vegetation variables, ranging from 50 m (no. of seedlings, no. of liana, dbh of large trees) to 460 m (height of large trees) (Additional file 1: Table S2, Fig. 2).

**Fig. 3:**
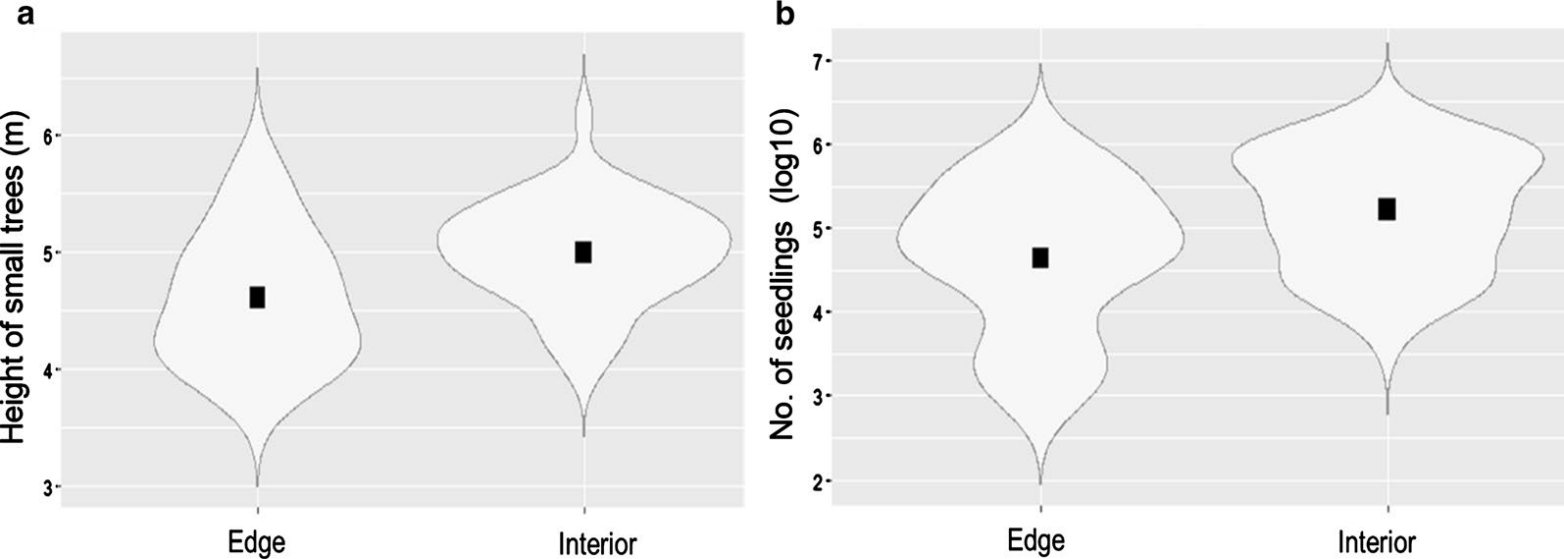
Comparison between edge and interior habitats for the two significant vegetation parameters: height of small trees (**a**) and number of seedlings (**b**). Black squares: mean, violin plots range from minimum to maximum value with violin width illustrating data distribution

The comparison between the interior and edge habitats for each vegetation parameter revealed only two significant differences (Additional file 1: Table S1, Additional file 2). First, small trees were significantly taller in the interior than in the edge (Estimate = 0.391, p = 0.032, Fig. 3a). Second, seedlings had a significantly higher density in the interior compared to edge habitat (Estimate = 0.573, p = 0.049, Fig. 3b).

### Effects of edge habitats on *M. ravelobensis* abundance and body mass

**Fig. 4:**
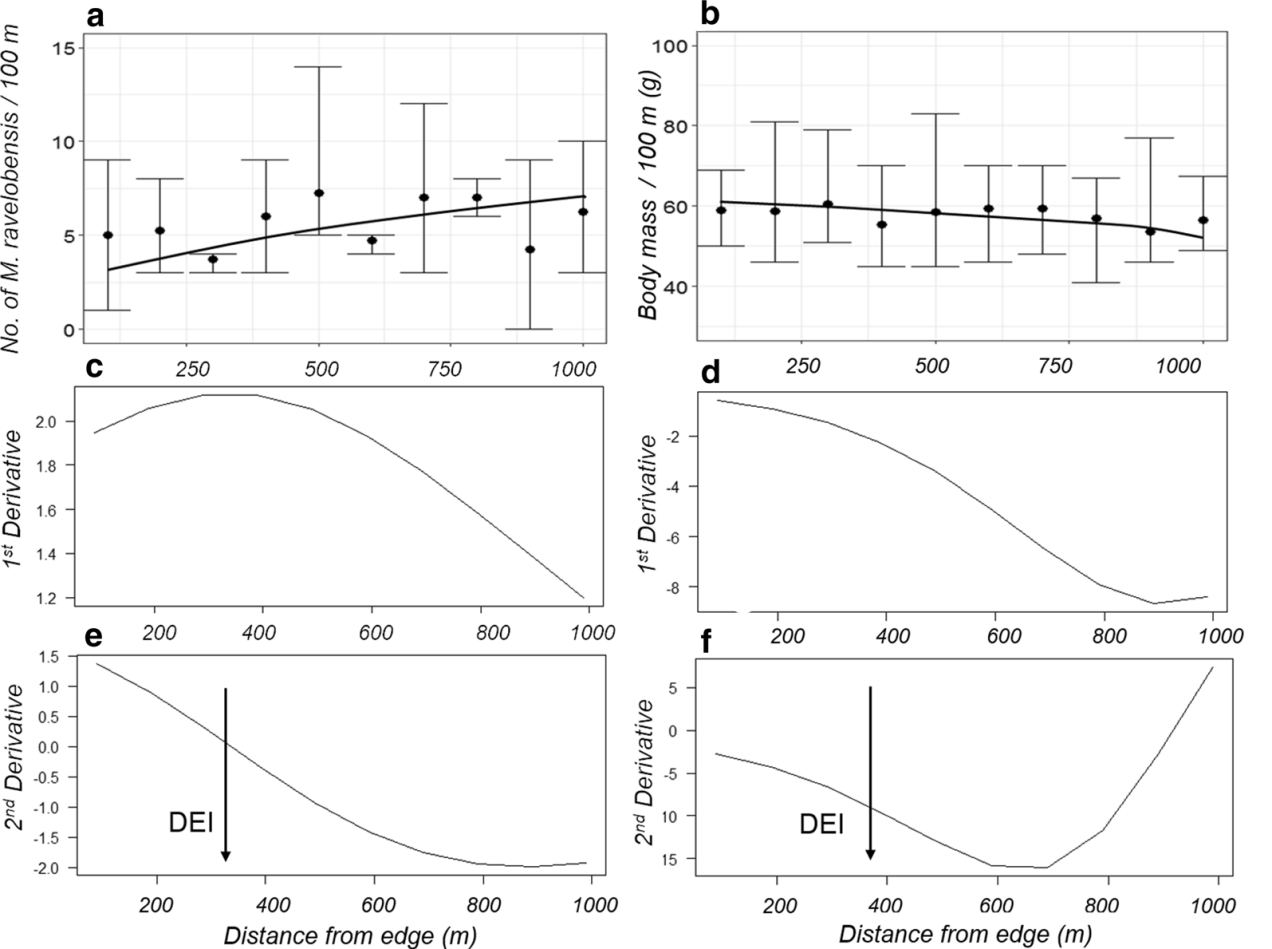
Variation of abundance (**a**) and body mass (**b**) of *M. ravelobensis* from the edge into the interior of forest and calculation of their DEI, respectively. Mean values (dots) are plotted together with minimum and maximum values (whiskers). In addition, the first derivatives of the fitted curves (**c**, **d**: abundance and body mass) and the second derivatives (e and f: abundance and body mass) are plotted. The DEI (vertical arrows in (**e**) for the abundance and in (**f**) for the body mass) is determined as the distance between the two first inflection points in the curve of the second derivative

The edge-dependent variation in the abundance of *M. ravelobensis* was best explained by a logistic function (R^2^ = 0.283, Fig. 4a, Suppl. Information 1 Tables S2, S3). The DEI for *M. ravelobensis* abundance was 340 m (Fig. 4a, c, e). Spatial variations in the body mass of *M. ravelobensis* were also best explained by the logistic function (R^2^ = 0.352, Fig. 4b, Additional file 1: Table S2, S3) and the DEI for changes in body mass was 390 m (Fig. 4b, d, f).

**Fig. 5:**
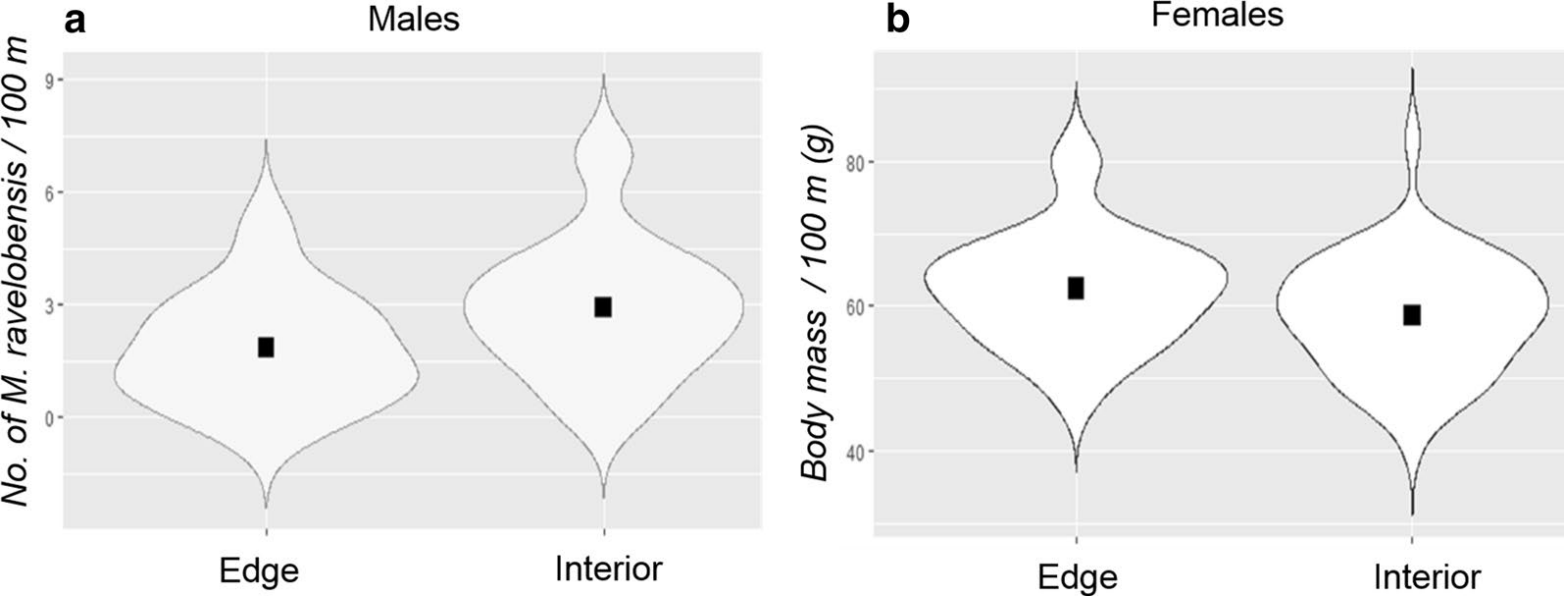
Comparison of **a** abundance (males only), and **b** body mass (females only) of *M. ravelobensis* between edge and interior habitats in form of violin plots. Black squares show the mean, violin plots end with minimum and maximum values

Overall, there was no significant difference in *M. ravelobensis* abundance between the interior and edge habitats. However, as a statistical trend, males but not females had a higher abundance in the interior than in the edge (Estimate_males_ = 1.083, n_edge_ = 22, n_interior_ = 70, p = 0.082, Estimate_females_ = − 0.039, n_edge_ = 34, n_interior_ = 74, p = 0.843, Fig. 5a, Additional file 1: Table S3, Additional file 2).

There was no significant difference in the overall body mass of *M*. *ravelobensis* between the edge and interior habitats, but, as a statistical trend, females but not males weighed more in edge than in interior habitats (Estimate = − 3.735, n_edge_ = 27, n_interior_ = 48, p = 0.052, Fig. 5b, Additional file 1: Table S3, Additional file 2).

### Relationship between edge effects in vegetation and *M. ravelobensis* abundance and body mass

Based on the comparison of the DEIs derived for the vegetation parameters and the abundance and body mass variations in *M. ravelobensis*, edge-related changes in the number of small trees corresponded best to changes in *M. ravelobensis* abundance and body mass (DEI_small trees_: 360 m, DEI_abundance_: 340 m, DEI_body mass_: 390 m).

**Fig. 6:**
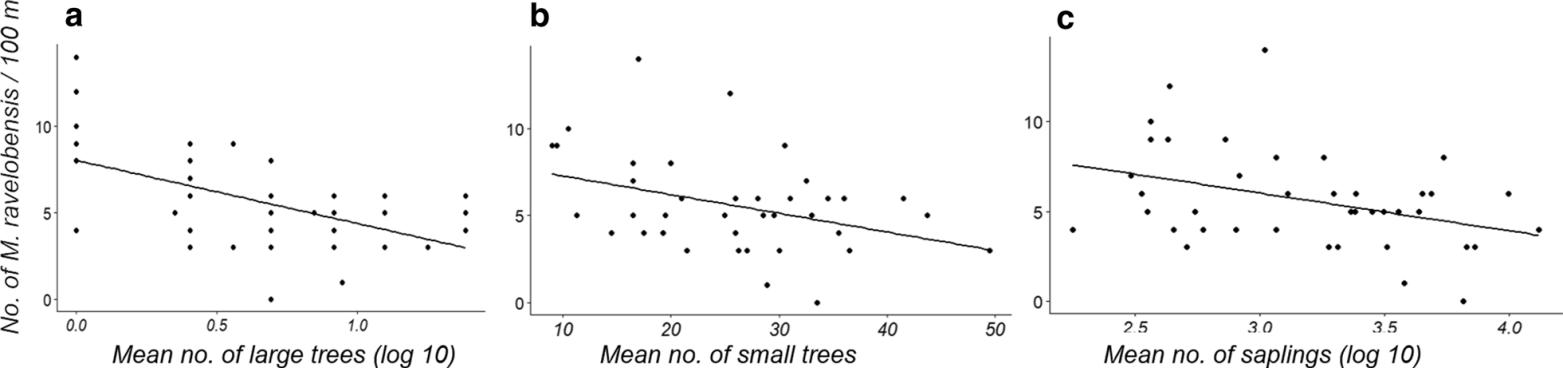
Relationship between three vegetation parameters (**a**–**c**: density of large, small trees and saplings, respectively) and the abundance of *M. ravelobensis*. Linear regression lines are shown for the three parameters

**Fig. 7:**
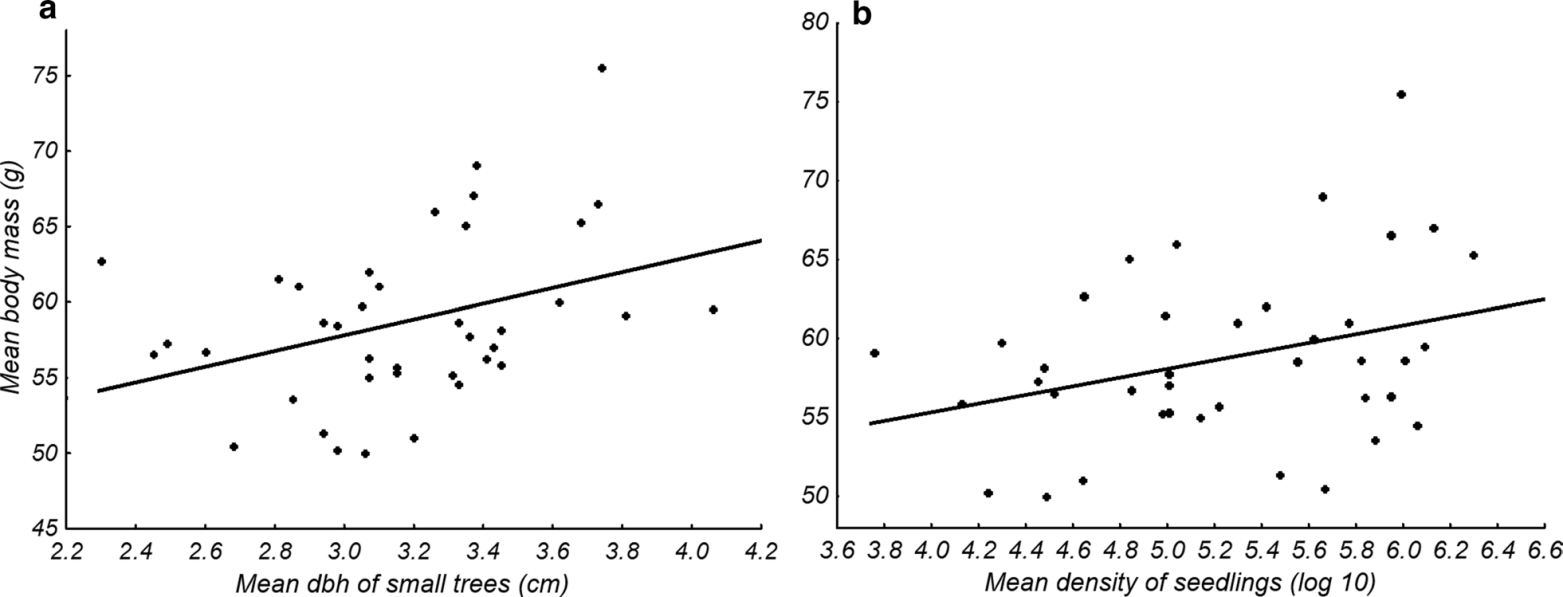
Relationship between three vegetation parameters (**a**, **b**: dbh of small trees and density of seedlings, respectively) and body mass of *M. ravelobensis*. Linear regression lines are shown for the two parameters

The potential relationship between the vegetation parameters and the abundance and body mass of *M. ravelobensis*, respectively, was analyzed by means of regression analyses (Additional file 1: Table S4, Additional file 3). These analyses revealed that the abundance of *M. ravelobensis* was best explained by the density of large trees (R^2^ = 0.279, n = 40, p < 0.001, Fig. 6a), but also, to a lesser extent, by the density of small trees (R^2^ = 0.141, n = 40, p = 0.017, Fig. 6b) and the density of saplings (R^2^ = 0.138, n = 40, p = 0.019, Fig. 6c). In all cases, the relationship between the variables was negative, i.e. the abundance of *M. ravelobensis* decreased with increasing density of large and small trees and with increasing density of saplings. None of the other variables explained variations in abundance. Significantly positive relationships were found between body mass and the dbh of small trees (R^2^ = 0.133, n = 40, p = 0.023, Fig. 7a) and the density of seedlings (R^2^ = 0.108, n = 40, p = 0.042, Fig. 7b, Additional file 1: Table S4).

## Discussion

### Edge effects on vegetation structure

Whereas edge effects for various vegetation parameters are reported quite regularly from different regions worldwide [e.g. 7, 8, 16], the depth of edge influence (DEI) is less often inferred statistically. A DEI was determined for all nine vegetation parameters in this study and was well supported in seven of nine variables (R^2^ > 0.1). In line with previous work in other ecosystems [17, 18, 48], the response pattern as well as the penetration depth varied considerably between parameters with the DEI varying from 50 m (for the density of seedlings, liana and dbh of large trees) to 460 m (height of large trees) into the forest. When comparing vegetation parameters between the edge and interior habitats, though, only two were significantly different. Specifically, the density of seedlings and the height of small trees were higher in the interior than in the edge habitats. This result was partly similar to the findings in the dry deciduous forest of Ankarafantsika NP [31], in the humid forests in Vohibola III in southeast Madagascar [26] and in a tropical rain forest in central Amazon forest where the density of seedlings increased towards the forest interior [9, 49]. These results may relate to the vulnerability of edge habitats to elevated levels of desiccation and wind speed, which can cause high rates of tree fall and tree mortality along edge habitats, which in turn can affect forest structure and composition [14]. For example, in the Gatún Lake central Panama topical forest, germination and seedling survival were lower due to wind-exposure in edge habitats compared to protected sites in the forest interior [50]. In addition, previous studies examined how fragmentation impacted the susceptibility of a fragmented boreal forest to wind damage. They found that wind speed negatively affected tree height near the edge [51, 52]. Thus, we suggest that the edge contrast, combined with spatial patterns of tree height and dbh, could operate synergistically to make trees more susceptible to wind damage at our research sites.

The lack of significant differences in the other seven vegetation variables between edge and interior habitats could be due to three explanations: (a) there was no systematic variation in these parameters along the transects, (b) inter-transect variations were higher than edge-interior variations, and (c) edge-interior differences exist but are only small and could not be detected due to the small sample size of four transects in our study. Whereas the first explanation may explain the poor modelling results for the number and dbh of large trees, it is unlikely for the other parameters, since substantial variation was detected in them (Fig. 2). Inter-transect variations were indeed often large (explanation (b), Fig. 2). A high variation between transects may arise if vegetation structure is not only determined by edge-interior dynamics but, for example, by varying levels of human disturbance on different transects or if different matrix types impact the adjacent forest in different ways [14]. Both aspects cannot be excluded for this study. The four transects differed in their distance to the village Mariarano, the largest settlements in the area, although smaller settlements of one to several houses were also scattered across the region (Fig. 1). Although the forest is under some basic level of protection, it is still used by the villagers for wood extraction, zebu grazing, charcoal production, or localized slash-and-burn activities to create new open spaces for agriculture and village enlargement. The resulting impacts may not have affected all transects equally. Moreover, all four transects bordered on different types of matrix, ranging from a dirt road over rice fields to savannah with different palm density (see methods). It is known that edge-related gradients in biotic variables are likely to be less pronounced when the structure of the matrix is similar to the original habitat [53], which may apply here to transects C1 (road with degraded forest on the other side of the road) and C4 (savannah with high palm density). Taken together, our study provided only limited evidence for systematic edge responses in vegetation structure and results may be best explained by local habitat divergence. Future studies with a repeated design will be needed to investigate the differential impact of the matrix on vegetation structure in Malagasy landscapes. These factors may act on both local but also on landscape levels in complex ways, as predicted by the ‘Landscape-Divergence Hypothesis’ [14], leading to divergent trajectories in vegetation structure and plant species assemblages even across various spatial scales.

### Edge effects on animal abundance and body mass

Forest-dwelling species can react in different ways and to a different extent to the presence, depth of penetration, and intensity of forest edges [54]. Our study in Mariarano revealed a DEI of 340 m on the abundance of *M. ravelobensis* and a DEI of 390 m on the body mass of *M. ravelobensis*. Comparative values of the DEI for mouse lemurs from other sites are not yet available, but these values are in the same range as a value estimated for the larger-bodied diurnal *Propithecus coquereli* (~ 400 m) [17].

A visual inspection of the relationship between edge distance and abundance or body mass in *M. ravelobensis* revealed only moderate edge effects, and we also did not find significant differences in the overall abundance and body mass of this species between edge and interior habitats. These results would be congruent with a rather “neutral” response of *M. ravelobensis* to edges [4]. When analyzing these relationships separately in both sexes, however, statistical trends emerged for a higher abundance of males but not females in the interior habitat, while females but not males weighed slightly more in the edge versus the interior habitat.

The abundance results are in stark contrast to those of a previous study on *M. ravelobensis* which reported a higher overall female abundance at the edge compared to the interior habitat in Ankarafantsika NP, which is only about 90 km away from Mariarano [33]. Two aspects may explain these different findings: First, the two Ankarafantsika NP sites bordered on the same homogenous open savannah matrix [33], whereas the four transects in Mariarano bordered on four different matrix types, which may have impacted vegetation composition and structure differently [53] and may have resulted in a more variable abundance dataset in Mariarano (Fig. 4a). Such an interpretation would also be in agreement with the heterogeneous findings on the vegetation structure (see above). Second, the forest sites from Ankarafantsika NP contained not only *M. ravelobensis* but also *M. murinus* in variable proportions (11.3% at edge, 60.9% in the interior) which was not the case in Mariarano with just one *M. murinus* among 226 captured *M. ravelobensis* individuals across all four 1000 m transects. Burke and Lehman (2013) consequently also discussed interspecific competition as one potential driver of edge-interior utilization patterns which can be excluded for our study.

Similar to our study, females but not males weighed significantly more in the edge compared to the interior habitat in Ankarafantsika NP [33]. The authors argued that *M. ravelobensis* may weigh more at the edge due to an increased access to insect food that was shown to be available in higher abundance at the edge than in the interior in the same study [33]. Further studies on habitat use and feeding ecology will be needed to understand potential sex differences in body mass in this species in edge and interior habitats.

### Relationship between vegetation structure, animal abundance and body mass

Various characteristics of vegetation structure have previously been suggested to determine habitat suitability for mouse lemurs and were proposed to be related to resource availability, efficient locomotion and shelter quality [55–59]. For example, *M. ravelobensis* occurred in higher abundance in sites with a high abundance of lianas, more and larger shrubs, a lower density of medium-sized trees (dbh: 5–10 cm), and overall in more humid habitats in Ankarafantsika NP [36, 40, 41]. Conversely, *M. ravelobensis* was found to be absent from forest habitats with smaller trees, a low density and low dbh of large trees, a low density of shrubs and with much open ground, characteristics that can all be directly or indirectly linked to dry conditions [36]. On the other hand, this species was also reported to possess a high microhabitat flexibility, as it was trapped in 19 out of 22 study sites (86.4%) within the continuous part of the Ankarafantsika NP [36].

When evaluating the congruence between the DEI’s of *M. ravelobensis* abundance and adult body mass and the DEI’s on the different elements of vegetation structure in this study, the animal DEI’s (340 m and 390 m) corresponded best to the DEI of 360 m for the density of small trees. This relationship was further supported by a negative correlation between *M. ravelobensis* abundance and the number of small trees per plot, although this parameter did not differ significantly between the edge and the interior habitat. In addition, *M. ravelobensis* abundance increased with a decreasing number of large trees and that of saplings (Fig. 6). Conversely, the body mass of *M. ravelobensis* increased with increasing dbh of small trees and density of seedlings, suggesting an impact of vegetation structure on mouse lemur abundance and body mass which may be partly independent of edge effects. The underlying reasons for these relationships, however, cannot be clarified at present, since they are not in line with previous findings from Ankarafantsika NP (see above), and cannot be easily linked to food resource availability, protection from predators, or suitable shelters for daily sleeping groups [38, 59, 60]. One limitation of this study was the lack of floristic data. Since plant parts such as fruits, nectar or gum as well as arthropods and insect secretions do belong to the consumed food items of *M. ravelobensis* [61], it is possible that the local distribution of some key plant/tree species or arthropods may have impacted the local abundance and body mass of this lemur species independently of potential edge/habitat gradients. Thus, future research on the ecological patterns underlying edge effects in animal species should ideally include concordant data on the spatial distribution, abundance and use of potential food resources. In doing so, results from these studies can be used to test and refine conceptual models on how resource distribution effects abundances and body mass near edge habitats [62].

## Conclusion

This study contributes to our knowledge of ecological edge effects on the vegetation structure in dry deciduous forests and revealed moderate but not very consistent depth of edge influences in various vegetation parameters. Likewise, such rather weak effects could also be identified in the abundance and body mass of the vulnerable *M. ravelobensis*. A comparison of our results with findings from a different study region revealed that edge effects seem to be highly variable on the local (between transects) as well as on the landscape level (between study regions) within the vegetation structure but also within the same study species, a small nocturnal primate from Madagascar. A high ecological flexibility of this lemur species towards variable ecological conditions at forest edges may explain such seemingly contradictory responses, and also its occurrence in several different types of microhabitats [36]. Such a flexibility may enable this species to cope with disturbances and some anthropogenic habitat changes within forests [63], although they seem to respond negatively to habitat fragmentation per se, probably due to a limited potential to connect between fragmented forest patches across open space [37]. Further studies are urgently needed to evaluate the critical features of landscapes that are needed to sustain viable populations of this vulnerable lemur species.

Depth of edge influences are rarely measured in a rigorous and interdisciplinary way with the simultaneous evaluation of vegetation and animal responses like attempted in this study. Edge effects are therefore not well understood for most taxonomic groups and for most parts of Madagascar, although forest loss is ongoing and omnipresent [23]. Our study detected signals of high local habitat divergence coupled with high regional landscape divergence in northwestern Madagascar. To fully understand such complex cases, even broader approaches are needed to integrate fine-scale abiotic and biotic measurements of edge effects in floristic and structural forest composition across multiple replicate sites at local and broad spatial scales. Only then the species-specific resilience towards different types of habitat disturbance, forest degradation, matrix effects, habitat loss and fragmentation can be fully disentangled. These insights are urgently needed, as they will ultimately guide conservation biologists and stakeholders to formulate efficient conservation guidelines for species such as the unique lemurs of Madagascar.

## Supplementary information

**Additional file 1: Table S1.** Variation of all vegetation parameters (per 40 m^2^) and the statistical comparison between edge and interior habitats according to their depth of edge influence (DEI) in Mariarano Classified Forest, northwestern Madagascar. **Table S2.** Comparison of the fit of four functions modeled for each vegetation and animal parameter in Mariarano Classified Forest, northwestern Madagascar. **Table S3.** Variation in abundance and body mass of all adult *Microcebus ravelobensis* and the comparison between edge and interior habitats after determining the respective depth of edge influence (DEI) in Mariarano Classified Forest, northwestern Madagascar. **Table S4.** Results of regression analyses between all nine vegetation parameters and abundance and body mass, respectively, of *Microcebus ravelobensis* in Mariarano Classified Forest, northwestern Madagascar.

**Additional file 2.** Dataset that was used for the statistical comparison of all vegetation and animal parameters (one value per double plot per transect if applicable) between the “edge habitats” and the “interior habitats”.

**Additional file 3.** Dataset that was used for the regression analyses between all vegetation parameters and the two animal parameters abundance and body mass (one value per 100 m per transect).

## Data Availability

All data analysed during this study are included in the supplementary information files to this published article. The datasets analysed during the current study are also available from the corresponding author on reasonable request.
