# Supplementary material for: Effects of habitat edges on vegetation structure and the vulnerable golden-brown mouse lemur (Microcebus ravelobensis) in northwestern Madagascar
Source: BMC Ecol. 2020 Dec 17;20:69. doi: 10.1186/s12898-020-00337-z (PMC7745463; doi:10.1186/s12898-020-00337-z)
Supplement: Supplementary file 1 — Additional file 1: Table S1. Variation of all vegetation parameters (per 40 m2) and the statistical comparison between edge and interior habitats according to their depth of edge influence (DEI) in Mariarano Classified Forest, northwestern Madagascar. Table S2. Comparison of the fit of four functions modeled for each vegetation and animal parameter in Mariarano Classified Forest, northwestern Madagascar. Table S3. Variation in abundance and body mass of all adult Microcebus ravelobensis and the comparison between edge and interior habitats after determining the respective depth of edge influence (DEI) in Mariarano Classified Forest, northwestern Madagascar. Table S4. Results of regression analyses between all nine vegetation parameters and abundance and body mass, respectively, of Microcebus ravelobensis in Mariarano Classified Forest, northwestern Madagascar. [file 12898_2020_337_MOESM1_ESM.docx]

| **Content of Supplementary material 1.docx** |
| --- |
|  |
| **Effects of habitat edges on vegetation structure and the vulnerable golden-brown mouse lemur (*Microcebus ravelobensis*) in northwestern Madagascar** |
|  |
| **Bertrand Andriatsitohaina^1^, Daniel Romero-Mujalli^2^, Malcolm S. Ramsay^3,2^, Frederik Kiene^2^, Solofonirina Rasoloharijaona^1,4^, Romule Rakotondravony^1,4^, Shawn M. Lehman^3^ and Ute Radespiel^2^** |
|  |
| **^1^ Ecole Doctorale Ecosystèmes Naturels (EDEN), University of Mahajanga, Madagascar** |
| **^2^ Institute of Zoology, University of Veterinary Medicine Hannover, Germany** |
| **^3^ Department of Anthropology, University of Toronto, Canada** |
| **^4^ Mention Sciences de la Vie et de l'Environnement, Faculté des Sciences, de Technologies et de l’Environnement, University of Mahajanga, Madagascar** |
|  |

**Table S1:** Variation of all vegetation parameters (per 40 m^2^) and the statistical comparison between edge and interior habitats according to their depth of edge influence DEI in Mariarano Classified Forest, northwestern Madagascar. Dist. (m): Distance from the edge in meter (position of double plots along perpendicular transects), EH: Edge habitat, IH: Interior habitat, Est. (p): Estimate value from generalized linear model (statistical error). The vertical bold lines indicate which plot data were summed up for the edge dataset (left side of the first bold line) and which were included in the interior dataset (right side of the second bold line); (in bold: significant differences when comparing the edge zone and interior zone by GLMs.

**Table S2**: Comparison of the fit of four functions modeled for each vegetation and animal parameter in Mariarano Classified Forest, northwestern Madagascar. AICc: Akaike’s Information Criterion corrected, β_1_, β_2_ and β_3_: Constants, DEI: depth of edge influence. (in bold: best function), na: not available

**Table S3:** Variation in abundance and body mass of all adult *Microcebus ravelobensis* and the comparison between edge and interior habitats after determining the respective depth of edge influence (DEI) in Mariarano Classified Forest, northwestern Madagascar. Dist.: Distance from the edge in segments of 100m along the perpendicular transects, EH: Edge habitat, IH: Interior habitat, Est. (p): Estimate value from Generalized linear model (statistical error), F: females, M: males. The vertical bold lines indicate points were summed up for the edge dataset (left side of the first bold line) and which were included in the interior dataset (right side of the second bold line) (in bold: statistical trend, p < 0.1).

**Table S4:** Results of regression analyses between all nine vegetation parameters and abundance and body mass, respectively, of *Microcebus ravelobensis* in Mariarano Classified Forest, northwestern Madagascar. (In bold: variables with significant regression results). *: p < 0.05, ***: p < 0.001

|  | Abundance (individuals/100m) | | | | Body mass (g) | | | |
| --- | --- | --- | --- | --- | --- | --- | --- | --- |
| Predictor | Estimate | SE | R^2^ | p-value | Estimate | SE | R^2^ | p-value |
| Intercept | 8.041 | 0.729 |  | < 0.001 | 57.597 | 1.702 |  | < 0.001 |
| Log mean no. of large tree | **-3.643** | **0.951** | **0.279** | **< 0.001***** | 1.727 | 2.215 | 0.016 | 0.441 |
| Intercept | 8.355 | 1.159 |  | < 0.001 | 56.592 | 2.471 |  | < 0.001 |
| Mean no. of small tree | **-0.107** | **0.043** | **0.141** | **0.017*** | 0.085 | 0.092 | 0.023 | 0.360 |
| Intercept | 4.329 | 0.765 |  | < 0001 | 59.099 | 2.170 |  | < 0.001 |
| Mean dbh of large trees | 0.043 | 0.045 | 0.027 | 0.354 | 0.010 | 0.128 | 0.000 | 0.937 |
| Intercept | 7.595 | 3.736 |  | 0.049 | 42.202 | 6.993 |  | < 0.001 |
| Mean dbh of small trees | -0.611 | 1.165 | 0.007 | 0.603 | **5.203** | **2.186** | **0.133** | **0.023*** |
| Intercept | 9.751 | 2.791 |  | 0.001 | 54.723 | 8.097 |  | < 0.001 |
| Mean height of large tree | -0.474 | 0.275 | 0.085 | 0.094 | 0.450 | 0.798 | 0.010 | 0.577 |
| Intercept | 3.948 | 5.287 |  | 0.460 | 49.411 | 10.541 |  | < 0.001 |
| Mean height of small tree | 0.340 | 1.053 | 0.003 | 0.748 | 1.866 | 2.104 | 0.021 | 0.381 |
| Intercept | 12.353 | 2.755 |  | < 0.001 | 51.735 | 5.907 |  | < 0.001 |
| log mean no. of saplings | **-2.107** | **0.856** | **0.138** | **0.019*** | 2.210 | 1.845 | 0.037 | 0.239 |
| Intercept | 7.112 | 3.598 |  | 0.055 | 44.336 | 6.871 |  | < 0.001 |
| log mean no. of seedlings | -0.278 | 0.679 | 0.004 | 0.685 | **2.748** | **1.302** | **0.108** | **0.042*** |
| Intercept | 5.186 | 1.072 |  | < 0.001 | 59.323 | 2.157 |  | < 0.001 |
| log mean no. of lianas | 0.351 | 0.738 | 0.006 | 0.637 | -0.447 | 1.477 | 0.002 | 0.764 |
